# Power of the policy: how the announcement of high-stakes clinical examination altered OSCE implementation at institutional level

**DOI:** 10.1186/1472-6920-13-8

**Published:** 2013-01-24

**Authors:** Chi-Wei Lin, Tsuen-Chiuan Tsai, Cheuk-Kwan Sun, Der-Fang Chen, Keh-Min Liu

**Affiliations:** 1 Department of Medical Education, E-Da hospital, I-Shou University, No.1, Yida Road, Yanchao Dist, Kaohsiung City, 82445, Taiwan; 2 Graduate Institute of Adult Education, National Kaohsiung Normal University, No.116, Heping 1st Rd., Lingya Dist, Kaohsiung City, 80201, Taiwan; 3 Department of Pediatrics, E-Da hospital, No.1, Yida Road, Yanchao Dist, Kaohsiung City 82445, Taiwan; 4 Department of Chinese Medicine, I-Shou University, No.1, Yida Road, Yanchao Dist, Kaohsiung City 82445, Taiwan; 5 Department of Surgery, Cathay General Hospital, No.280 Renai Rd. Sec.4, Taipei City, Taiwan; 6 Fu-Jen Catholic University College of Medicine, No.510, Zhongzheng Rd., Xinzhuang Dist, New Taipei City, Taiwan; 7 Department of Anatomy, College of Medicine, Kaohsiung Medical University, No. 100, Shih-Chuan 1st Road, Kaohsiung City, 80708, Taiwan

**Keywords:** OSCE, Assessment, Policy, High-stakes

## Abstract

**Background:**

The Objective Structured Clinical Examination (OSCE) has been widely applied as a high-stakes examination for assessing physicians’ clinical competency. In 1992, OSCE was first introduced in Taiwan, and the authorities announced that passing the OSCE would be a prerequisite for step-2 medical licensure examination in 2013. This study aimed to investigate the impacts of the announced national OSCE policy on implementation of OSCE at the institutional level. Further, the readiness and the recognition of barriers toward a high-stakes examination were explored.

**Methods:**

In 2007 and 2010, the year before and after the announcement of high-stakes OSCE policy in 2008, respectively, questionnaires on the status of OSCE implementation were distributed to all hospitals with active OSCE programs in Taiwan. Information on OSCE facilities, equipment, station length, number of administrations per year, and the recognition of barriers to the success of implementing an OSCE were collected. The missing data were completed by telephone interviews. The OSCE format, administration, and facilities before and after the announcement of the nationwide OSCE policy were compared.

**Results:**

The data were collected from 17 hospitals in 2007 and 21 in 2010. Comparing the OSCE formats between 2007 and 2010, the number of stations increased and the station length decreased. The designated space and the equipment for OSCE were also found to have been improved. As for the awareness of OSCE implementation barriers, the hospital representatives concerned mostly about the availability and quality of standardized patients in 2007, as well as space and facilities in 2010.

**Conclusions:**

The results of this study underscored an overall increase in the number of OSCE hospitals and changes in facilities and formats. While recruitment and training of standardized patients were the major concerns before the official disclosure of the policy, space and facilities became the focus of attention after the announcement. The study results highlighted the influence of government policy on different aspects of OSCE implementation in Taiwanese training institutes that showed high level of support as reflected in the improved hardware and the change in OSCE format to serve the summative purpose.

## Background

Since the first attempt of implementing the Objective Structured Clinical Examination (OSCE) in the University of Dundee of the United Kingdom in 1975 [1], it has been accepted as an important assessment tool for evaluating the clinical skills of medical students and healthcare professionals. The first national licensure OSCE was adopted by the Medical Council of Canada (MCC) in 1992 [2], and later by many other countries, such as the United States [3] and Korea [4,5].

The OSCE was initially introduced in Taiwan in early 1990s [6] and soon accepted as a kind of formative assessment in several medical schools and teaching hospitals [7]. Due to an increased focus on physicians’ clinical competencies, in 2008, the authorities in Taiwan announced that passing the OSCE would be a prerequisite for taking the written test of clinical science medical licensure examination (i.e. step 2) in 2013. The national examination will be a 12-station audiovisual recorded OSCE held in multiple test sites. To ensure the success of the high-stakes OSCE in 2013, two large-scale pilot OSCEs were performed nationwide in 2011 and 2012.

Previous studies on OSCE mainly focused on experience-sharing [8-10], the reliability in terms of psychometrics [11-13], and review of the validity of OSCE after its implementation [14,15]. On the other hand, the implementation of high-stakes OSCE is a formidable challenge to all medical educators, especially those in countries where OSCE is still in its infancy in the medical education system. This study, therefore, aimed at investigating the impact of the announcement of high-stakes OSCE on its preparation in terms of hardware and software, format, and awareness at major clinical educational institutes and medical centers in Taiwan, thereby shedding light on the socio-political influences on the assessment of clinical competencies in medical education and the consequences.

## Methods

There were 130 and 129 institutes qualified as teaching hospitals in 2007 and 2010, respectively (i.e. the year before and after the announcement of the initiation of national high-stakes OSCE in 2008). Purposive sampling was performed through distributing questionnaires on the status of implementation of OSCEs to all of the teaching hospitals with active OSCE programs during the two respective time periods, the list of which had been acquired through internet surfing and telephone confirmation. All questionnaires were filled in by the personnel responsible for OSCE administration who were supposed to thoroughly understand all details about OSCE implementation at their own institutes. Data missing from incomplete questionnaires were then collected through telephone. The information providers were not required to identify themselves or provide personal opinions. The questions covered information on OSCE facilities, equipment, station length, and the frequency of administrations each year.

To investigate the recognized barrier to OSCE success at an institutional level, another part of the questionnaire for each year covered information on the key components of OSCE implementation, including standardized patient, raters, data analysis, space and equipment, case writing, and administration. A 5-point Likert-type scale was given to each item: 1 - Remarkable difficulty hampering OSCE implementation; 2 - Major obstacle requiring time-consuming efforts to overcome; 3 - Minor difficulty not significantly hindering OSCE implementation; 4 - Fairly smooth implementation; 5 - Implementation with notable success. Recognized barrier to success of implementing OSCE was defined as an item to which the representative of the institute gave a score of 1 or 2.

The data acquired before and after the announcement of the nationwide OSCE were regarded as coming from a distinct group in each year and examined for differences by chi-square test, but if the corresponding expected value was less than 5 in any one cell of the related 2x2 table, Fisher’s exact test was used instead to obtain the p-value. A p-value <0.05 is considered statistically significant.

The questionnaire was designed to collect data at the institutional level on an anonymous basis without involving expression of personal opinions. The whole study was reviewed by the Institutional Review Board (IRB) of Clinical Research Ethics of E-Da Hospital which granted exemption from IRB review for the present study.

## Results

Of the 17 and 23 hospitals with active OSCE programs in 2007 and 2010, respectively, the questionnaire response rates were 100% (17/17) in 2007 and 92.3% (21/23) in 2010. All the medical school-affiliated teaching hospitals had been included in this survey because of their hosting OSCE for either training or assessing the clinical competency of medical students in both years.

### Implementation format

There was no significant fluctuation in the proportion of hospitals that ran OSCEs at least 4 times a year between 2007 and 2010 (64.7% in 2007 and 61.9% in 2010). On the other hand, six hospitals (35.3%) had more than 9 stations for each OSCE session in 2007, while the number of hospitals with 9 stations or more increased to 13 (61.9%) in 2010. As for the station length, 40% of the hospitals had regular-length station of 4-11 min in 2007, whereas the number of hospitals increased to 14 (66.7%) in 2010. Despite the apparent changes in the implementing format of the OSCE between 2007 and 2010 as reflected in the increase in the number of stations for each OSCE session and the preferred adoption of regular-length station instead of long station, they failed to reach statistical significance (Table 1).

**Table 1:** Comparison of the differences of formats and facilities in high-stakes OSCE in Taiwan between 2007 and 2010

**Item**	**2007**	**2010**	**p**
	**(n = 17)**	**(n = 21)**	
Frequency ≧4 times/year	11 (64.7%)	13 (61.9%)	0.86
Stations ≧9 per track	6 (35.3%)	13 (61.9%)	0.10
Station length 4-11 min	7 (41.2%)	14 (66.7%)	0.11
Holding OSCE in standard test rooms	9 (52.9%)	16 (76.2%)	0.13
All rooms with AV recording system	5 (29.4%)	15 (71.4%)	0.01*

Note: *p < 0.05 indicating the significance.

Abbreviation: *AV *Audio-visual.

### Space and facilities

Compared with those in 2007, the facilities and equipment for OSCE in the surveyed institutes were found to have improved in 2010. In 2007, only 9 (52.9%) hospitals had built standard test rooms for running OSCE programs, and that number increased to 16 (76.2%) in 2010. The increase, however, was not statistically significant (Table 1). On the other hand, in 2007, while only 5 hospitals (29.4%) were equipped with audiovisual recording systems in all the test rooms, that number tripled to 15 (71.4%) in 2010, showing significant improvement (*p* = 0.01) in hardware installation between the two years (Table 1).

### Awareness of barriers toward excellence

The awareness of implementation barriers toward excellence also showed some differences between these two years. In 2007, the most important concerns of the hospitals were recruitment and training of standardized patients (SPs) (7 hospitals, 41.2%), while the main issues became space and facilities (10 hospitals, 47.6%), followed by the preparation of raters (7 hospitals, 33.3%), and case writing ability (6 hospitals, 28.6%) in 2010. The aggravated concern regarding the need for space and facilities was statistically significant (p = 0.02), whereas the other changes in items of awareness were insignificant between the two years (Table 2).

**Table 2:** The awareness of barriers encountered in high-stakes OSCE in Taiwan between 2007 and 2010

**Item**	**2007**	**2010**	**p**
	**(n = 17)**	**(n = 21)**	
Standardized patient	7 (41.2%)	4 (19.0%)	0.10
Raters	4 (23.5%)	7 (33.3%)	0.23
Data analysis	3 (17.6%)	4 (19.0%)	0.32
Space and equipment	2 (11.8%)	10 (47.6%)	0.02*
Case writing	2 (11.8%)	6 (28.6%)	0.15
Administration	1 (5.9%)	1 (4.8%)	0.51

Note: *p < 0.05 indicating the significance.

No. and proportion indicate the ones with data ≧4 in a 5-point Likert-type scale.

## Discussion

A high-stakes educational assessment procedure, such as a national OSCE, and the related official requirements may influence different aspects of the routine implementation at clinical education institutes that used to utilize OSCE as a formative educational tool. In Taiwan, there had been little change in the test format of OSCE during the initial 15 years, from its debut in 1992 to the survey in 2007. This study showed that the official announcement of high-stakes OSCE as an upcoming national examination played a catalytic role in facilitating OSCE standardization at institutional level.

The essential features of an OSCE of high quality include a well-planned blueprint with multi-faceted tasks and multi-stations and well-trained SPs and raters [16]. It has been reported that an increase in the length of OSCE, a careful selection and an increase in the number of stations (>10), as well as a combination of OSCE and other less resource-intensive methods, such as a written test, can increase the reliability of OSCE scores [17,18].

In 2007, 17 clinical training institutes claimed to be able to run OSCEs regularly for medical students. With the emphasis on assessment for learning, the OSCEs were characterized by a small number of long stations. Due to facility and space limitation, examinations were performed mainly in clinical areas with only a limited number of stations being held in standard test rooms. Beside facilities, other essential components that constitute an OSCE for summative purpose were also missing.

Immediately after the official announcement of OSCE as part of the medical licensure examination by the Ministry of Education in Taiwan, preparation for the high-stakes assessment tool was started at different medical education institutes. Within three years from 2007 to 2010, the number of training hospitals capable of running regular OSCEs for medical students increased by 41.2% (from 17 to 23) with a concomitant rise in the number of stations and curtailing of station length. Besides, the number of institutes with designated test rooms for OSCE increased from 9 (52.9%) in 2007 to 16 (76.2%) in 2010. Furthermore, the number of hospitals with audiovisual equipment was substantially elevated from 5 (29.4%) to 15 (71.4%) (*p* = 0.01), highlighting an overall increase in the number of institutes with active OSCE programs and significant improvement mainly in facilities of test rooms following a decision of augmenting financial investment in compliance with the criteria of accreditation for national test centers of high-stakes OSCE.

Although the number of stations for each track, the duration of each station, and the use of standardized test rooms tended to follow the summative format, there was no significant difference in the number of institutes that ran OSCE at a high frequency (i.e. ≧4). Indeed, the number of such institutes was higher in 2010 than in 2007. The findings imply a stage of preparation in response to the policy through retaining the high frequency of examination not only to familiarize the trainees with the format of high-stakes OSCE as well as to provide experience for potential raters and SPs, but also to accumulate experience at an institutional level. Taken together, all the major clinical educational institutes in Taiwan showed willingness to improve the OSCE quality toward a high-stakes level, and to offer OSCE practices for their trainees.

Differences were also noted between the two years in the recognition of difficulties encountered during OSCE implementation. In 2007, the recruitment and training of SPs were the major concerns, while space and facilities, as well as case writing were the major weaknesses perceived in 2010. The differences may be explained by the formative nature of OSCE in 2007 with strong emphasis being placed on communication skills, physical examination, and professional behaviors. Besides, SPs were assigned the duty of providing quality feedbacks for candidates in scenarios which were commonly complex and embedded with emotions. SPs were asked to develop fine performance skills and to be well articulate. Since SPs were the key determinants of a successful learning experience, training SPs was highlighted as a difficult task. On the other hand, after the announcement of the OSCE policy in 2010, competing for accreditation became a major concern. However, spaces were mainly utilized for clinical service in most clinical training centers without adequate room designated for educational assessment that requires sophisticated audiovisual recording system. Space and facilities, therefore, became the most important concerns for the education institutes. Moreover, the establishment of the bank of case scenarios for high-stakes OSCE required high-quality contributions from faculty members of each institute that imposed another challenge to the training centers (Table 2).

The limitation of this study is the difficulty in completely excluding the impact of time as a potential confounder in the study observations rather than the policy announcement itself. However, since little change had been observed after the implementation of OSCE during the past 15 years before 2007, it is rational to believe that the improvement by leaps and bounds in facilities between 2007 and 2010 could be attributed to the announcement of the high-stakes OSCE policy.

## Conclusions

The results of this study underscored an overall increase in the number of hospitals capable of running OSCE and significant improvement in facilities after announcement of the high-stakes OSCE policy. Some of the formats including number of stations and station length were also changed despite the lack of statistical significance. While recruitment and training of SPs were the major concerns before the official disclosure of the policy, space and facilities gained most attention after the announcement. The results of this study indicate a high level of support from the medical training institutes in Taiwan toward the implementation of the upcoming high-stakes OSCE as reflected in the significant improvement in hardware, the change in OSCE format to the summative direction, and the high frequency of OSCE training at the medical education institutes to familiarize themselves with the high-stakes format. The recognized difficulties described in this survey may serve as a reference for medical educators toward implementation of high-stakes OSCE.

## Abbreviations

OSCE: Objective structured clinical examination; SPs: Standardized patients.

## Competing interests

The authors declare that they have no competing interests.

## Authors’ contributions

CWL designed the study, participated in the data acquisition and analysis, and drafted the manuscript. TCT participated in the study design, interpreted the data, and revised the manuscript critically. CKS participated in analysis and interpretation of data, and drafted the manuscript. DFC participated in the data acquisition and revised the manuscript critically. KML participated in the study design and coordination, and revised the manuscript critically. All authors read and approved the final manuscript.

## Pre-publication history

The pre-publication history for this paper can be accessed here:

http://www.biomedcentral.com/1472-6920/13/8/prepub
